# Henoch-Schönlein purpura with epididymo-orchitis: A rare extra-cutaneous manifestation

**DOI:** 10.1016/j.eucr.2024.102919

**Published:** 2024-12-25

**Authors:** Majd Oweidat, Islam H. Karajeh, Widad Abu Mayyala, Mohammed Alra'e, Ammar Y. Hmidat

**Affiliations:** a Department of Surgery, College of Medicine, Hebron University, Hebron, Palestine; b Department of Pediatrics, Princess Alia Hebron Governmental Hospital, Hebron, Palestine; c Department of Pediatrics, College of Medicine, Hebron University, Hebron, Palestine; d Department of Pediatrics, Al-Ahli Hospital, Hebron, Palestine

**Keywords:** Henoch-schönlein purpura, iga vasculitis, Epididymo-orchitis, Testicular involvement

## Abstract

Henoch-Schönlein Purpura (HSP), or IgA vasculitis, is a systemic inflammatory disorder primarily affecting children. While common symptoms include purpura, abdominal pain, and arthritis, testicular involvement is rare. We report a case of a young boy presenting with severe scrotal pain and swelling, later identified as epididymo-orchitis secondary to HSP. Diagnosis was confirmed via Doppler ultrasound, and treatment with corticosteroids and antibiotics resulted in rapid symptom resolution. This case highlights the importance of recognizing rare extra-cutaneous manifestations of HSP and underscores the need for prompt diagnosis and tailored management to achieve favorable outcomes.

## Highlights

• Epididymo-orchitis as a rare manifestation of Henoch-Schönlein Purpura (IgA vasculitis).
• Doppler ultrasound aids in distinguishing orchitis from testicular torsion in vasculitis.
• Corticosteroid therapy resolves severe scrotal symptoms and reduces inflammation.
• Case underscores vigilance for atypical presentations in pediatric systemic vasculitis.
• Early diagnosis and tailored treatment ensure favorable outcomes in rare IgA vasculitis cases.

## 1 Introduction

Henoch-Schönlein purpura (HSP), also known as IgA vasculitis (IgAV), is a systemic inflammatory disorder caused by the deposition of immune complexes in the walls of small blood vessels. This leads to inflammation and subsequent leakage of blood. It is the most common cause of vasculitis in the pediatric population, affecting 10 to 20 per 100,000 children per year.^1^ The peak incidence of IgAV occurs in children between the ages of 4 and 7, although it can affect individuals of any age, with a slight male predominance.^2^

Numerous risk factors have been linked to the development of IgAV, but the exact mechanisms by which these factors induce the disorder remain unknown. Genetic and environmental factors, such as infections, immunizations, insect bites, foods, and drugs, are believed to elicit this abnormal immune response.^3^

The clinical manifestations of IgAV can vary, but the most common features include skin rash, gastrointestinal tract involvement, arthritis, and IgA nephropathy. Involvement of other organs, such as the central nervous system (CNS), lungs, and testicles, is rare.^4^ Testicular involvement in IgAV is relatively uncommon, with an estimated incidence of 1.3 % in male patients.^5^

While the clinical manifestations of IgAV are well-documented, rare complications such as epididymo-orchitis are less frequently reported, potentially leading to delays in diagnosis and management. Documenting cases like this is crucial to expanding the understanding of IgAV's full clinical spectrum and guiding timely, evidence-based interventions for atypical presentations.

Diagnosis of IgAV is based on the characteristic rash and one or more additional features, such as abdominal pain, arthritis, renal involvement (evidenced by urine analysis), histological evidence of IgA deposition in the glomeruli (as proliferative glomerulonephritis) or blood vessels (as leukocytoclastic vasculitis).^6^ Scrotal tenderness, and swelling are often observed on examination, and Doppler ultrasound may reveal decreased blood flow. IgA levels are elevated in up to 40 % of cases.^7^ Additionally, patients with scrotal involvement in IgAV tend to have lower serum IgA levels compared to those without this manifestation. Treatment in these cases may be conservative, medical (using steroids), or surgical.^8^

This case underscores the rarity and diagnostic complexity of epididymo-orchitis as a manifestation of HSP. Scrotal involvement occurs in male IgAV patients, and isolated epididymo-orchitis are exceedingly uncommon.^9^ This unique presentation adds to the limited pool of cases and highlights the necessity for heightened clinical suspicion when scrotal pain occurs alongside systemic vasculitis symptoms.

## 2 Case presentation

A previously healthy boy in his early childhood presented with diffuse, colicky abdominal pain, which was followed by a palpable purpuric rash on both lower extremities. The rash began on the dorsum of his feet and gradually spread to his knees and thighs. His family sought medical advice at an outpatient clinic, where he was diagnosed with HSP and managed as an outpatient (Table 1).

**Table 1:** The child’s initial laboratory results.

	Test	Result	Normal Range
Urine analysis (UA)	Color	Yellow Straw	
	Turbidity	Turbid	
	RBCs	1–2	0- 5 HPF
	WBCs	1–2	0-5 HPF
	Bacteria	Few	0-10 HPF
	protein	Negative	
Hematology	WBC	11.7 ∗10^3^	(5.5–15.5)∗10^3^ cell/L
	RBC	4.65	3.9–5.3 cell/L
	НЬ%	11.6	10.5–14 g/dL
	MCV	78.1	70-74 fl
Special Tests	Creatinine	0.43	0.7–1.2 mg/dl
	BUN	12	5–18 mg/dl
	AST	20	0–50 U/L
	ALT	22	0–41 U/L

Two days later, he presented to our emergency department with severe scrotal pain and swelling. This was accompanied by tactile fever, joint pain, swelling, and limping. The patient throat was clear, and there was no history of nausea, vomiting, bloody stool, hematuria, scrotal trauma, or urinary tract infections.

**Fig. 1:**
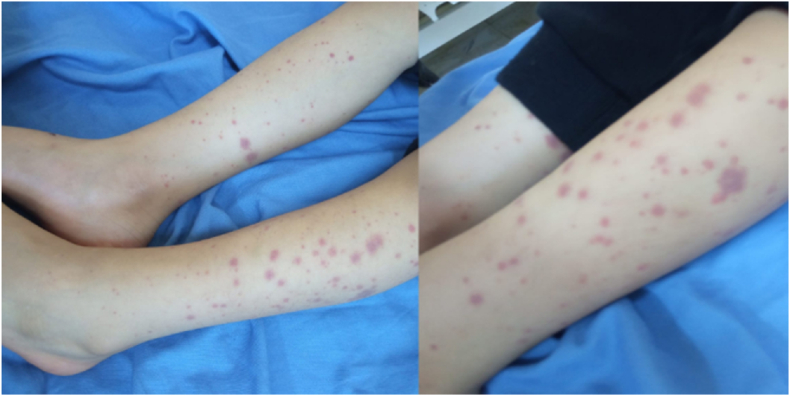
Palpable purpuric rash on the lower extremities. The rash is diffuse, polymorphic, maculopapular, and non-blanchable, with a characteristic red-to-purple coloration. The rash started over the dorsum of both feet then gradually spread up till the knees, thighs and buttocks.

On arrival, the child appeared to be in pain, but his vital signs were within normal ranges. Physical examination revealed a petechial and purpuric rash diffusely distributed over both feet, extending to the knees and thighs. The rash was palpable, maculopapular, polymorphic, non-blanchable, and red to purple in color. His abdomen was soft and non-tender, as demonstrated in Fig. 1. His chest was clear on auscultation, with good bilateral air entry and no additional sounds.

**Fig. 2:**
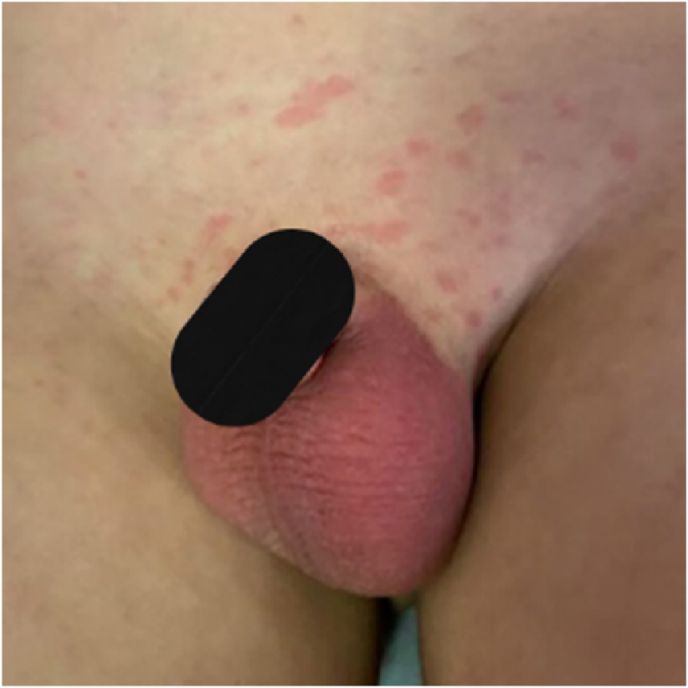
A photograph reveals significant erythema and edema of the scrotal wall.

Genital examination showed an erythematous and edematous scrotal wall that was tender to palpation. The pain was not alleviated by scrotal elevation, as shown in Fig. 2. Doppler ultrasound of the scrotum revealed an enlarged left epididymis and spermatic cord, both of which were edematous and heterogeneous with increased vascularity on color Doppler. Additionally, there was slightly increased vascularity in the left testicle with a surrounding mild to moderate reactionary hydrocele. The scrotal wall was markedly edematous. The right testicle was located in the inguinal canal and appeared normal in size, shape, and echotexture. These findings were consistent with left-sided epididymitis-funiculitis with possible early orchitis.

The child was admitted for close monitoring and treatment. The following treatment regimen was initiated: intravenous Ampicillin (500 mg every 12 hours), intravenous Gentamicin (25 mg every 8 hours), oral Ibuprofen, and Methylprednisolone (2 mg/kg/day divided into two doses over 4 hours for three days). Methylprednisolone was subsequently transitioned to oral Prednisolone with tapering.

**Fig. 3:**
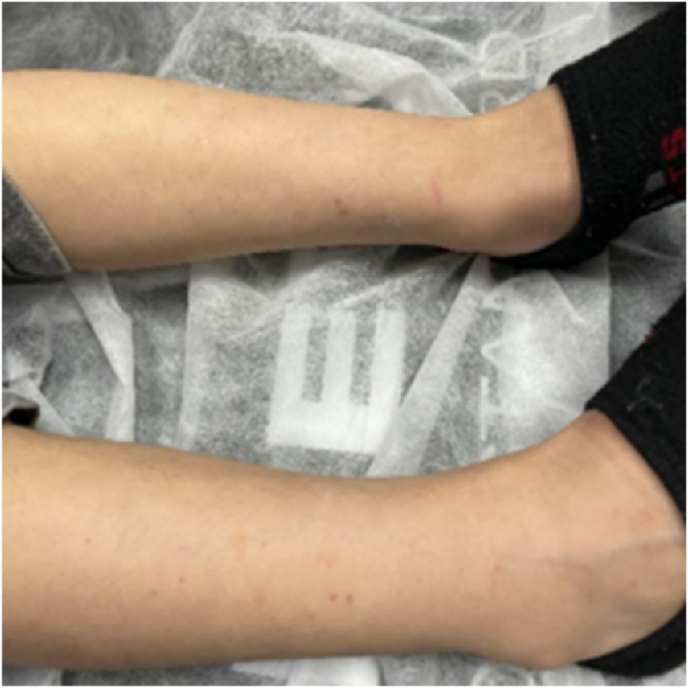
Resolution of the lower extremities purpuric rash following treatment.

The child's condition improved during his hospital stay. The rash faded significantly, as illustrated in Fig. 3. His joint pain and abdominal discomfort resolved. A follow-up Doppler ultrasound performed four days after admission demonstrated marked improvement in the previously noted epididymitis-funiculitis, with reduced size and edema. However, the left testicle displayed a relatively small, ill-defined, faint hypoechoic area measuring approximately 3 × 2 mm, warranting follow-up.

At four weeks, the previously described diffuse edematous scrotal wall was no longer evident. The right testicle appeared normal in size, shape, echotexture, and position. The child was discharged after five days of hospitalization and was prescribed oral Prednisolone (1.5 mg/kg) for an additional five days. Unfortunately, one day after completing steroid therapy, the rash reappeared on the buttocks. Laboratory investigations were performed, and the results, shown in Table 2, demonstrated normal ranges for complete blood count, kidney function, liver function tests, and urinalysis.

**Table 2:** The child’s laboratory results one day after completing steroid therapy.

	Test	Result	Normal Range
Urine analysis (UA)	Color	Straw	
	Turbidity	Turbid	
	RBCs	1–2	0- 5 HPF
	WBCs	1–2	0-5 HPF
	Bacteria	Few	0-10 HPF
	Protein	Negative	
Hematology	WBC	15.5 ∗10^3^	(5.5–15.5)∗10^3^ cell/L
	RBC	4.46	3.9–5.3 cell/L
	НЬ%	11.7	10.5–14 g/dL
	MCV	76.9	70-74 fl
	MCH	26.2	27–31.2
Special Tests	Creatinine	0.39	0.7–1.2 mg/dl
	BUN	10	5–18 mg/dl
	AST	21	0–50 U/L
	ALT	24	0–41 U/L

The treatment plan included restarting prednisolone (1.5 mg/kg) for five days, followed by tapering. A follow-up ultrasound was recommended after four weeks.

## 3 Discussion

The exact cause of IgAV remains unclear, although certain trigger events, such as upper respiratory infections—particularly those caused by group A β-hemolytic streptococcus—are commonly associated with its onset. IgA, especially the IgA1 subclass, plays a central role in the disease's development. Abnormal glycosylation in the hinge region of IgA1 makes it more susceptible to recognition by autoantibodies, leading to the formation of immune complexes. After the deposition of those complexes in small blood vessels of various organs, they trigger inflammation through complement activation. Clinically, the hallmark symptoms of IgAV include palpable purpura (seen in all cases), joint pain or swelling (66–74 %), gastrointestinal symptoms (51–56 %), and renal involvement (30–54 %).^10^ The rash in IgAV is typically painless, non-blanchable, non-pruritic, and occurs without thrombocytopenia or coagulopathy, classically appearing on the lower limbs with an ascending pattern. Gastrointestinal symptoms include abdominal pain, nausea, and vomiting, but bleeding and intussusception can also occur. Arthritis generally affects the lower limbs, although other joints may also be involved. Renal involvement can lead to hematuria and proteinuria.^6^

In children with IgAV, scrotal involvement presents with symptoms such as pain, redness, and swelling, often without urinary issues.^9^ Testicular blood vessels can serve as targets of systemic vasculitis due to immune complex deposition, leading to vascular inflammation and increased permeability in scrotal tissues. The resulting swelling and pain are linked to immune-inflammatory responses, including oxidative stress, cytokine release, and increased vascular permeability.^11^

IgAV in males can present with various scrotal and testicular symptoms, including swelling and pain in the scrotum, spermatic cord, and testis, as well as conditions like epididymitis, epididymo-orchitis, hematomas around the testis, and even testicular torsion. The incidence of these manifestations ranges from 2 % to 38 %.^12^ Other less common genitourinary complications of IgAV include urethritis with hydronephrosis, ureteral calcification, bladder wall hematomas, and hemorrhage in the spermatic cord.^13^ Rarely, patients may also experience priapism, axial purpura, hemorrhagic cystitis, renal colic, ureteral calcification, bladder hematomas, and urethral stricture, though these symptoms generally resolve on their own.^14^

Scrotal involvement frequently manifests as epididymitis (67 %) or epididymo-orchitis.^15^ Ultrasound is the primary diagnostic tool, often showing increased blood flow or inflammation of the scrotum. Notably, inflammatory markers such as white blood cell (WBC) count, C-reactive protein (CRP), and erythrocyte sedimentation rate (ESR) are typically normal or elevated. Increased d-dimer levels, reflecting a hypercoagulable state, are a sensitive marker for acute thrombosis and may correlate with renal complications in IgAV.^16^

A review of 21 case reports from 1986 to 2020 provided valuable insights into IgAV cases with scrotal involvement. The average age of onset for children with scrotal symptoms was 5.69 ± 2.12 years. The majority presented with scrotal pain accompanied by redness and swelling, typically without urinary difficulties. Scrotal involvement often coincided with other systemic manifestations, including gastrointestinal symptoms (48 %), joint involvement (42 %), fever (19 %), and hematuria (9 %). Rarely, penile involvement was reported in 9 % of cases. Regarding the timing of scrotal symptoms relative to IgAV diagnosis, scrotal involvement occurred after the onset of IgAV in 67 % of cases, prior to the onset in 24 %, and simultaneously in 9 %. This variability suggests that scrotal involvement may appear at any stage of the disease. The relationship between scrotal involvement and renal or joint complications remains unclear, with conflicting evidence. Some studies suggest an association between scrotal and renal involvement, while others indicate no clear connection or temporal relationship.^15^

IgAV without renal involvement is generally managed conservatively using analgesics and anti-inflammatory medications. In cases of complications, glucocorticoids are typically administered. Nonsteroidal anti-inflammatory drugs (NSAIDs) are commonly prescribed to address arthritis symptoms. For patients with renal involvement, treatment often includes a combination of glucocorticoids, immunosuppressive agents, and Angiotensin-converting enzyme (ACE) inhibitors.^17^^,^^18^ According to the literature, a systematic review article showed that most cases of IgAV typically require only supportive care and pain management, corticosteroids may be necessary for certain complications. In addition to IgAV nephritis, corticosteroids are considered for severe manifestations such as orchitis, cerebral vasculitis, pulmonary hemorrhage, and significant gastrointestinal involvement^19^. We administered steroids to the patient in this case after reviewing the literature and examining similar cases. Surgical exploration is reserved for cases where testicular torsion cannot be excluded or complications such as necrosis arise. Monitoring d-dimer levels may guide prognosis, as elevated levels are associated with renal injury risks.^15^ The prognosis of HSP is typically favorable, though recurrence is frequently observed in children, with rates ranging from 2.7 % to 66.2 %.^20^ Additionally, the recurrence of symptoms following steroid withdrawal is notable, as it required tailored adjustments to the therapeutic regimen. This reinforces the importance of individualized treatment plans and close follow-up in managing HSP with atypical features.

This case aligns with the literature in that scrotal involvement occurred after other IgAV symptoms, such as purpura and gastrointestinal issues, highlighting the need to consider rare complications like epididymo-orchitis in pediatric patients with scrotal pain. This presentation illustrates the diagnostic challenges associated with distinguishing epididymo-orchitis from conditions such as testicular torsion, which often leads to diagnostic uncertainty and the potential for unnecessary surgical interventions. Doppler ultrasound, as utilized in this case, is crucial for differentiating between these conditions, facilitating timely and appropriate management. Additionally, the recurrence of symptoms, such as rash following steroid withdrawal, underscores the variability in the disease course and the importance of individualized management plans. While clinical evidence links HSP with epididymo-orchitis, the exact mechanisms remain unclear. Immune complex deposition, vascular inflammation, and predisposition to scrotal involvement are not fully understood. This case also reinforces the importance of heightened clinical suspicion for rare extra-cutaneous manifestations of IgAV, contributing to better recognition and management strategies. The absence of genetic or immunological studies limits insights into potential susceptibilities. Future research should investigate genetic or immunologic factors that predispose to scrotal involvement in IgAV, and explore biomarkers to deepen understanding of the disease's pathophysiology. Longitudinal studies on scrotal involvement could provide valuable insights into recurrence and management.

## 4 Conclusion

This case highlights the importance of recognizing rare extra-cutaneous manifestations of HSP, particularly epididymo-orchitis. The patient's rapid improvement with conservative treatment underlines the effectiveness of corticosteroids and antibiotics in managing scrotal involvement in IgAV. Clinicians should maintain vigilance for such very rare presentations, as timely diagnosis and appropriate management are crucial for favorable outcomes. Future studies are needed to explore the genetic and immunological mechanisms behind scrotal involvement in IgAV and to develop more targeted diagnostic and therapeutic strategies.

## Funding

The author(s) received no financial support for the research, authorship, and/or publication of this article.

## CRediT authorship contribution statement

**Majd Oweidat:** Writing – review & editing, Writing – original draft, Validation, Supervision, Software, Project administration, Data curation, Conceptualization, Resources, Visualization. **Islam H. Karajeh:** Writing – original draft, Resources, Software, Validation, Visualization. **Widad Abu Mayyala:** Writing – original draft, Resources, Software, Validation, Visualization. **Mohammed Alra'e:** Writing – original draft, Conceptualization, Resources, Software, Validation, Visualization. **Ammar Y. Hmidat:** Visualization, Validation, Resources, Supervision, Conceptualization, Data curation, Software.

## Informed consent

Written informed consent was obtained from the patient's parents/legal guardian for publication and any accompanying images. A copy of the written consent is available for review by the Editor-in-Chief of this journal on request.

## Ethical approval

This is a case report. Therefore, it didn't require ethical approval from ethics committee.

## Declaration of competing interest

The author(s) declared no potential conflicts of interest with respect to the research, authorship, and/or publication of this article.

## Abbreviations

IgAV –: IgA vasculitis
HSP –: Henoch-Schönlein purpura
CNS –: Central nervous system
NSAIDs –: Nonsteroidal anti-inflammatory drugs
WBC –: White blood cell
CRP –: C-reactive protein
ESR –: Erythrocyte sedimentation rate
ACE –: Angiotensin-converting enzyme
UA –: Urine analysis
RBCs –: Red blood cells
HPF –: High-power field
Hb% –: Hemoglobin percentage
MCV –: Mean corpuscular volume
BUN –: Blood urea nitrogen
AST –: Aspartate aminotransferase
ALT –: Alanine aminotransferase

## References

[1] Hetland L., Susrud K., Lindahl K., Bygum A. (2017). Henoch-schönlein purpura: a literature review. Acta Derm Venerol.

[2] Audemard-Verger A., Pillebout E., Guillevin L., Thervet E., Terrier B. (2015). IgA vasculitis (Henoch–Shönlein purpura) in adults: diagnostic and therapeutic aspects. Autoimmun Rev.

[3] Yang Y.H., Yu H.H., Chiang B.L. (2014). The diagnosis and classification of Henoch–Schönlein purpura: an updated review. Autoimmun Rev.

[4] Oni L., Sampath S. (2019). Childhood IgA vasculitis (Henoch schonlein purpura)—advances and knowledge gaps. Front Pediatr.

[5] Hu J.J., Zhao Y.W., Wen R. (2023). Immunoglobulin a vasculitis with testicular/epididymal involvement in children: a retrospective study of a ten-year period. Front Pediatr.

[6] Trnka P. (2013). H enoch– S chönlein purpura in children. J Paediatrics Child Health.

[7] Jennette J.C., Falk R.J., Bacon P.A. (2012). Revised international chapel hill consensus conference nomenclature of vasculitides. Arthritis Rheum.

[8] Buscatti I.M., Abrão H.M., Kozu K. (2018). Characterization of scrotal involvement in children and adolescents with IgA vasculitis. Adv Rheumatol.

[9] Hara Y., Tajiri T., Matsuura K., Hasegawa A. (2004). Acute scrotum caused by Henoch-Schönlein purpura. Int J Urol.

[10] Peru H., Soylemezoglu O., Bakkaloglu S.A. (2008). Henoch Schonlein purpura in childhood: clinical analysis of 254 cases over a 3-year period. Clin Rheumatol.

[11] Xiang J., Cao K., Dong Y.T. (2020). Lithium chloride reduced the level of oxidative stress in brains and serums of APP/PS1 double transgenic mice via the regulation of GSK3β/Nrf2/HO-1 pathway. Int J Neurosci.

[12] Modi S., Mohan M., Jennings A. (2016). Acute scrotal swelling in henoch-schonlein purpura: case report and review of the literature. Urology Case Reports.

[13] Clark W.R., Kramer S.A. (1986). Henoch-Schönlein purpura and the acute scrotum. J Pediatr Surg.

[14] Davol P., Mowad J., Mowad C.M. (2006). Henoch-Schönlein purpura presenting with orchitis: a case report and review of the literature. Cutis.

[15] Ma Y., Zhang S., Chen J., Kong H., Diao J. (2021). Henoch-schönlein purpura with scrotal involvement: a case report and literature review. J Pediatr Hematol Oncol.

[16] Lindner G., Funk G.C., Pfortmueller C.A. (2014). D-dimer to rule out pulmonary embolism in renal insufficiency. Am J Med.

[17] Bluman J., Goldman R.D. (2014). Henoch-Schönlein purpura in children: limited benefit of corticosteroids. Can Fam Physician.

[18] Bista S., Adhikari Y., Karmacharya S., Joshi S., Pandey S., Adhikari N. (2023). Henoch–Schönlein purpura with antecedent allergic diseases in a 4-year-old child: a case report. Annals of Medicine & Surgery.

[19] Ozen S., Marks S.D., Brogan P. (2019). European consensus-based recommendations for diagnosis and treatment of immunoglobulin A vasculitis—the SHARE initiative. Rheumatology.

[20] Alfredo C.S., Nunes N.A., Len C.A., Barbosa C.M.P., Terreri M.T.R.A., Hilário M.O.E. (2007). Henoch-Schönlein purpura: recurrence and chronicity. J Pediatr (Rio J).

